# Transcriptomic Biomarkers for Tuberculosis: Validation of *NPC2* as a Single mRNA Biomarker to Diagnose TB, Predict Disease Progression, and Monitor Treatment Response

**DOI:** 10.3390/cells10102704

**Published:** 2021-10-09

**Authors:** Leonardo S. de Araujo, Marcelo Ribeiro-Alves, Matthew F. Wipperman, Charles Kyriakos Vorkas, Frank Pessler, Maria Helena Féres Saad

**Affiliations:** 1 Cellular Microbiology Laboratory, Oswaldo Cruz Institute (IOC), Oswaldo Cruz Foundation (FIOCRUZ), Rio de Janeiro 20045-360, Brazil; LdeAraujo@fz-borstel.de; 2 Research Group Biomarkers for Infectious Diseases, TWINCORE Centre for Experimental and Clinical Infection Research, 30519 Hannover, Germany; 3 National Institute of Infectology Evandro Chagas, Oswaldo Cruz Foundation (FIOCRUZ), Rio de Janeiro 21040-360, Brazil; mribalves@gmail.com; 4 Immunology Program, Memorial Sloan Kettering Cancer Center, New York, NY 10021, USA; Matthew.wipperman@gmail.com (M.F.W.); Charles.vorkas@stonybrookmedicine.edu (C.K.V.); 5 Clinical and Translational Science Center, Weill Cornell Medicine, New York, NY 10021, USA; 6 Division of Infectious Diseases, Renaissance School of Medicine, Stony Brook University, Stony Brook, NY 11794, USA; 7 Centre for Individualised Infection Medicine, 30625 Hannover, Germany; 8 Helmholtz Center for Infection Research, 38124 Braunschweig, Germany

**Keywords:** biomarkers, diagnosis, mRNA, *Mycobacterium tuberculosis*, Niemann–Pick disease type C2, *NPC2*, RNA, transcription, treatment, tuberculosis

## Abstract

External validation in different cohorts is a key step in the translational development of new biomarkers. We previously described three host mRNA whose expression in peripheral blood is significantly higher (*NPC2*) or lower (*DOCK9* and *EPHA4*) in individuals with TB compared to latent TB infection (LTBI) and controls. We have now conducted an independent validation of these genes by re-analyzing publicly available transcriptomic datasets from Brazil, China, Haiti, India, South Africa, and the United Kingdom. Comparisons between TB and control/LTBI showed significant differential expression of all three genes (*NPC2^high^* *p* < 0.01, *DOCK9^low^* *p* < 0.01, and *EPHA4^low^* *p* < 0.05). *NPC2^high^* had the highest mean area under the ROC curve (AUROC) for the differentiation of TB vs. controls (0.95) and LTBI (0.94). In addition, *NPC2* accurately distinguished TB from the clinically similar conditions pneumonia (AUROC, 0.88), non-active sarcoidosis (0.87), and lung cancer (0.86), but not from active sarcoidosis (0.66). Interestingly, individuals progressing from LTBI to TB showed a constant increase in *NPC2* expression with time when compared to non-progressors (*p* < 0.05), with a significant change closer to manifestation of active disease (≤3 months, *p* = 0.003). Moreover, *NPC2* expression normalized with completion of anti-TB treatment. Taken together, these results validate *NPC2* mRNA as a diagnostic host biomarker for active TB independent of host genetic background. Moreover, they reveal its potential to predict progression from latent to active infection and to indicate a response to anti-TB treatment.

## 1. Introduction

Tuberculosis (TB) is a curable infectious disease that remains a serious health problem worldwide, mainly due to inadequate diagnosis and treatment of infected individuals. Although concentrated in the poorest population groups, *Mycobacterium tuberculosis* (Mtb), the etiological agent of TB, may infect anyone. The complexity of Mtb control is clearly reflected by TB disease statistics: there are an estimated 10 million new cases yearly, one-third of the global population are latently infected with Mtb (LTBI), and there are 1.2 million deaths per year. In addition, drug-resistant TB strains remain a public health threat with around half a million cases worldwide [1]. Thus, a reduction in the global impact of TB requires improved diagnostic methods in order to identify both latently and actively infected individuals as early as possible, as well as tools to monitor treatment outcome. Mtb infection spans the spectrum between LTBI and active TB, and a given patient may be anywhere along this spectrum depending on the interplay between immune response and bacillary activity, which makes diagnosis even more complex. Tests based on the host’s immune response, such as tuberculin skin test (TST) and interferon gamma (INF-γ) release assays (IGRAs), although offering some help in clinical practice in the detection of LTBI, have important limitations. They do not distinguish LTBI from active TB among latently infected individuals, they do not indicate who is at greater risk of progressing to active disease (especially in populations with a high bacillary load), and they are not accurate in immunosuppressed individuals [2,3,4,5]. Moreover, aside from the complicated and time-consuming microbiological culture, there are no other tools available to monitor treatment.

Considering these shortcomings, there have been intense efforts to identify biomarkers that could serve as more accurate diagnostics and help to stratify individuals along the above-mentioned spectrum of TB. RNA biomarkers have received great attention in this regard because they often accurately reflect the regulation of processes underlying disease development and progression [6,7,8,9].

In our previous study of whole blood transcriptomes from Brazilian patients with latent and active TB, we identified three mRNA, *NPC2*, *EPHA4*, and *DOCK9*, with the highest AUROC (≥0.94) for TB in comparison to LTBI/controls. In the same previous study, we expanded the cohort and carried out a confirmatory analysis of these genes using quantitative polymerase chain reaction ((RT)qPCR). This confirmatory analysis showed that (at 92% specificity) sensitivity was highest for *NPC2* (85%) and less promising for *EPHA4* (53%) and *DOCK9* (19%) [10]. These three mRNA candidates achieved the optimal specificity (>80%) recommended for a community-based triage or referral test to identify people suspected of having TB according to the World Health Organization’s (WHO) TB target product profile (TPP). However, only *NPC2* approached the minimum TPP threshold for sensitivity (>90%) [9]. Moreover, confounders such as the broad clinical spectrum of TB infection and suboptimal detection of LTBI [5] might have limited the accuracy of those results. Interestingly, in a longitudinal analysis of a small number of samples, expression of *EPHA4* (*p* = 0.0003) and *NPC2* (*p* = 0.004) correlated significantly with a clinical response to anti-TB treatment [10], suggesting that the full biomarker potential of these genes was not completely investigated in our previous studies [10,11], and that *NPC2*, *EPHA4*, and *DOCK9* should be further evaluated in order to validate our initial findings.

The biological roles of *EPHA4* and *DOCK9* mRNA have not been completely elucidated. Previous studies suggest that both DOCK9 [12] and EPHA4 [13] proteins can interact with the Rho GTPase CDC42. Among other functions, Rho GTPases are described to regulate cytoskeletal kinetics and signal transduction pathways, playing a key role in the coordination of immune responses, including in the activation of T cells [14]. 

NPC1/NPC2 is a crucial pathway of intracellular cholesterol trafficking. While the protein NPC2, the product of the *NPC2* gene, is a soluble cholesterol-binding luminal protein, NPC1 is an anchored transmembrane glycoprotein [15,16,17]. In the lumen of late endosomes/lysosomes NPC2 transfers cholesterol to NPC1, then NPC1 transfers cholesterol to other vesicular pathways, e.g., endoplasmic reticulum [15,16,17]. Using an in vitro *M. tuberculosis* infection model, Wheelwright et al. (2014) [18] showed that supplementation with vitamin A, or all-trans retinoic acid (ATRA), contributed to the acidification of the lysosome, decreased intracellular cholesterol, altogether with a negative effect on mycobacterial viability. This mechanism was described as *NPC2*-dependent, up-regulated after the infection of phagocytes by *M. tuberculosis*, but the same was not the case with the NPC1 protein [18]. Indeed, *NPC1* mRNA was later shown not to be differentially expressed in the blood of TB patients [10].

In the present work, we performed an extensive evaluation of *NPC2, EPHA4*, and *DOCK9* mRNA levels (i) as diagnostic biomarkers according to WHO TPP criteria for a community-based triage or referral test to identify people suspected of having TB, (ii) as potential biomarkers for predicting progression from latent TB infection to active disease [19], and (iii) as correlates of a clinical response to anti-TB treatment. For this purpose, we analyzed previously published and unpublished datasets from cross-sectional tuberculosis cohorts from Brazil, Haiti, India, South Africa, and the United Kingdom, as well as from two prospective studies from China and South Africa.

## 2. Materials and Methods

### 2.1. Ethics Statements

The Brazilian study was approved by the Ethics Committee of the Oswaldo Cruz Foundation under registration code 560-10 [10]. Details about sample collection and ethical procedures of the Haitian cohort were previously published [20,21]. The data from the other cohorts were publicly available at GEO [22].

### 2.2. Terminology

We used the following case definitions and abbreviations. Control = healthy uninfected individuals recently exposed to a TB index case or not (the control subjects recruited by de Araujo et al. [10,11] and Wipperman et al. [20,21] were known to have been exposed to a TB index case but TB infection was subsequently ruled out). Symptomatic non-TB (S-NTB) = symptomatic adults self-presenting for investigation of pulmonary tuberculosis and showing no laboratorial evidence of active TB disease, regardless of the history of known exposure to a TB index case (for more details please check [6]). LTBI = defined by a positive Mantoux TST and/or IGRA and absence of active TB diagnostic [5]. TB = active tuberculosis diagnosed by sputum smear and/or culture and/or GeneXpert MTB/RIF [6,7,10,11,23,24,25]. TBtt = drug treatment for TB [7,24]. OD = other non-TB pulmonary diseases, such as active (aSARC) and non-active sarcoidosis (naSARC), lung cancer (LC), and pneumonia (PN) [23].

### 2.3. Inclusion Criteria for Eligible Published Datasets

The following search keywords were used to identify eligible datasets on GEO and ArrayXpress: human, transcriptomic, tuberculosis, and blood. Datasets that were deposited until March 2020 and were not present in our previous study [10] were included in this re-analysis. Inclusion criteria were studies containing the following characteristics. Biological specimens: whole blood or peripheral blood mononuclear cells (PBMC); subjects: adults (≥18 years old) with active pulmonary TB, LTBI, and controls with or without other diseases; transcriptomic profiling by RNAseq or microarray analysis. Exclusion criteria were samples from subjects <18 years of age or positive HIV status and non-human samples.

Table 1 summarizes the included cohorts. The Brazilian cohort is composed of a joint analysis of sub-cohorts from our two previous studies. In the first, we had originally discovered *NPC2*, *DOCK9*, and *EPHA4* mRNA as potential biomarkers for TB [10]. The second was focused on small noncoding RNA (sncRNA) expression in a larger group of samples [11], but we also used some of them to extract the normalized mRNA expression values for the present study. One unpublished dataset containing a cohort of Haitians and publicly available datasets from India were also included, following the criteria detailed above. Using all five cross-sectional cohorts, we evaluated expression of the three mRNA in TB patients from different geographic areas, also for the differentiation from non-TB pulmonary infections. In the longitudinal cohorts (two public datasets from China and South Africa), we evaluated their expression (i) in the progression from LTBI to active TB and (ii) during follow-up of anti-TB treatment.

For the Brazilian and Haitian cohorts, peripheral whole blood was collected in Paxgene RNA tubes (PreAnalytiX, SWZ) and processed and analyzed as described previously [10].

### 2.4. Acquisition and Normalization of Datasets

Transcriptomic data (microarray or RNAseq) of whole blood or PBMC were obtained as follows. GEO2R web tool [27] was used to gather normalized expression values of microarray studies. For RNAseq data, the FASTQ files were exported to the GREIN tool [28] and submitted to standard normalization. Processed expression data of E-MTAB-8290 [6] were downloaded from the ArrayExpress platform (https://www.ebi.ac.uk/arrayexpress/experiments/E-MTAB-8290/?page=1&pagesize=250, accessed on 31 March 2020) and included in our analysis. The normalized microarray or RNAseq expression values were exported to Prism 6 (GraphPad Software, 6.07, San Diego, CA, USA) for statistical analysis.

### 2.5. Statistical Analysis

Significance of differences between two groups was assessed with the Mann–Whitney (cross-sectional) or Wilcoxon (longitudinal) test. For comparisons of >2 groups, the Kruskal–Wallis (cross-sectional) or Friedman test (longitudinal) was used. Means, medians, standard deviations (SD), dispersion plots, area under the receiver operating characteristics curve (AUROC) values, 95% confidence intervals (CI), and coefficient of variation (CV) were computed using Prism 6 (GraphPad Software).

## 3. Results

### 3.1. Cross-Sectional Studies: Group Comparisons and ROC Analysis

#### 3.1.1. TB Detection

##### Studies Comparing with Control and LTBI

Cross-sectional published datasets were included in the re-analysis, which comprise subjects enrolled between 2009 and 2013 in India and the United Kingdom (London). Together with the Brazilian cohort enrolled from 2010 to 2013 and the Haitian cohort collected from 2016 to 2020, four cohorts from four different countries were, thus, available for analysis. In a first step, we assessed the diagnostic biomarker potential of *DOCK9, EPHA4*, and *NPC2* mRNA to distinguish among active TB, LTBI, and control groups.

**Figure 1:**
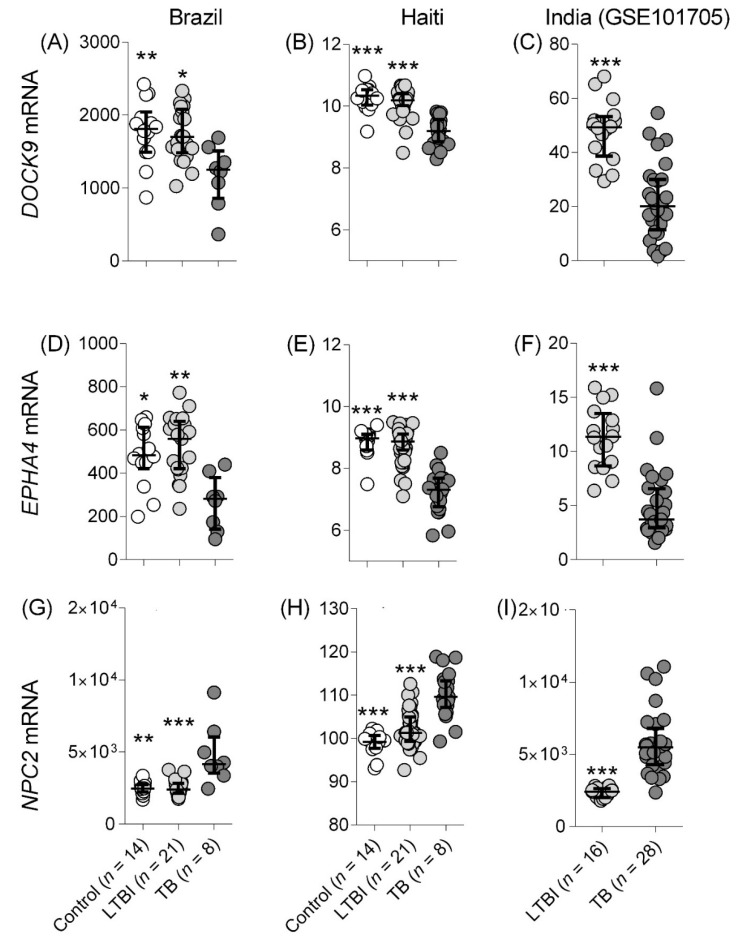
Individual normalized mRNA expression values of *DOCK9, EPHA4*, and *NPC2* mRNA in blood. Diagnostic groups comprise controls, latent tuberculosis infection (LTBI), and active tuberculosis (TB). (**A**–**C**) *DOCK9* mRNA expression in cohorts from Brazil (**A**), Haiti (**B**), and India (**C**). (**D**–**F**) *EPHA4* mRNA expression in cohorts from Brazil (**D**), Haiti (**E**), and India (**F**). (**G**–**I**) *NPC2* mRNA expression in cohorts from Brazil (**G**), Haiti (**H**), and India (**I**).The Mann–Whitney test was used to assess significance between 2 groups. The Kruskal–Wallis test was used to assess significance of differences across more than 2 groups, followed by Dunn’s multiple comparison tests correction. * *p*-value < 0.05; ** *p*-value < 0.01; *** *p*-value < 0.005 with respect to TB.

In accordance with our previous findings [10], active TB induced significantly higher *NPC2* mRNA levels and lower expression of *DOCK9* and *EPHA4* mRNA in the cohorts from Haiti and India (Figure 1). An *NPC2^high^* expression pattern (similar to the one observed among TB cases) was more frequent among LTBI than controls in the cohorts from Haiti (Figure 1H) and Brazil (Figure 1G), whilst LTBI from the Indian cohort showed less dispersed expression (Figure 1I). The control group showed a more heterogeneous expression profile of *DOCK9* and *EPHA4*, i.e., greater dispersion along the y-axis, which is more noticeable among the Brazilian (compare Figure 1A,D with Figure 1G) and Indian (compare Figure 1C,F with Figure 1I) cohorts. Overall, this analysis showed (i) that expression of all three mRNA changed in a similar fashion in blood of TB cases from the different geographic areas and (ii) that LTBI cases were more likely than non-infected samples to exhibit the “TB-like” pattern *NPC2^high^*.

ROC analysis of the distinction control vs. TB revealed high AUROC values (indicating accurate classification) with low variation for all three mRNAs among the different cohorts (*DOCK9*: 0.8–0.96; *EPHA4*: 0.89–0.97; *NPC2*: 0.91–0.99). Comparisons between latent and active infection also showed a similarly low variation of AUROC values (*DOCK9*: 0.86–0.95; *EPHA4*: 0.91–0.96; *NPC2*: 0.8 –0.98). This suggests that the high accuracy of these markers is reproducible among these ethnically different populations from diverse geographical locations.

In line with our previous findings [10], in this new reanalysis, *NPC2* showed the highest AUROC values for TB vs. non-TB discrimination among Brazilians (AUROC, TB vs. control/LTBI: 0.94), which was validated in the Indian (TB vs. LTBI: 0.98) as well as the British (TB vs. control: 0.99) cohort. Intriguingly, a slightly lower performance of *NPC2* (AUROC, TB vs. LTBI = 0.89) to differentiate TB vs. LTBI compared to *DOCK9* (TB vs. LTBI = 0.95) and *EPHA4* (TB vs. LTBI = 0.96) was observed in the Haitian cohort. As in high TB-burden settings, the chances of infection and progression to disease are higher, this might indicate that some subjects could actually be in the initial stages of progression, which could interfere with the performance of *NPC2^high^* as a biomarker for the binary distinction TB vs. LTBI (Figure 1).

Overall, the biomarker potential of *NPC2^high^* was successfully validated in these new analyses, which showed the highest mean AUROC values in the comparisons between TB and control (mean AUROC = 0.95) or LTBI (mean AUROC 0.94, Table 2). Further analysis will be performed in the next sections to obtain the sensitivity and specificity values of *NPC2^high^* for TB detection across the different cohorts included here. *DOCK9* and *EPHA4* also showed interesting biomarker value in this context.

##### Identification of TB in Individuals Presenting with Respiratory Symptoms

**Figure 2:**
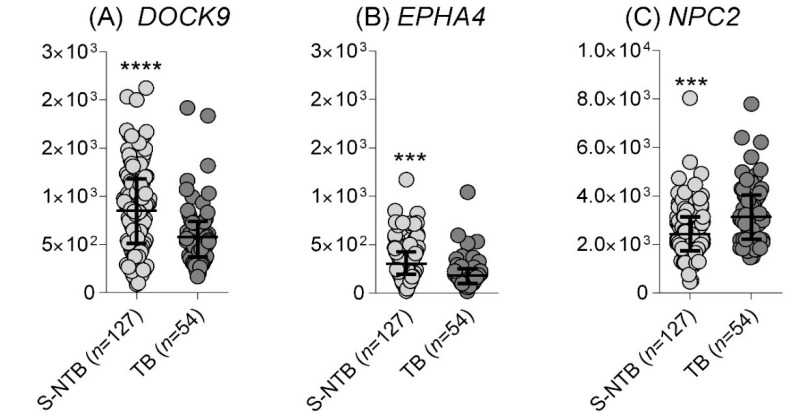
Individual normalized expression values of (**A**) *DOCK9*, (**B**) *EPHA4,* and (**C**) *NPC2* mRNA in blood in a South African cohort (E-MTAB-8290). The diagnostic groups comprise adults with respiratory symptoms self-presenting for investigation of pulmonary TB who were ultimately diagnosed as having TB or not. S-NTB: symptomatic adults showing no laboratorial evidence of active TB disease, regardless of the history of known exposure to a TB index case. TB = active tuberculosis. The Mann–Whitney test was used to assess significance between two groups: *** *p*-value < 0.005, **** *p*-value < 0.001.

Data from whole blood of individuals with respiratory symptoms, self-presenting for investigation of pulmonary TB, were available from the South African cohort [6]. Even though all subjects had respiratory symptoms, those diagnosed with TB by positive sputum smear and/or sputum liquid culture and/or GeneXpert TB/RIF test showed significant differences in *DOCK9* (*p*-value = 0.0002), *EPHA4* (*p* < 0.0001), and *NPC2* (*p* < 0.0001) mRNA expression when compared with non-TB subjects, as defined by negative results by the aforementioned diagnostic tests (Figure 2). However, all three mRNA were only moderately accurate in classifying the subjects into TB and non-TB cases: *EPHA4* showed a slightly higher AUROC (0.71, 95% CI: 0.63 to 0.79) than *NPC2* (0.68, 95% CI: 0.60 to 0.77) or *DOCK9* (0.675, 95% CI: 0.59 to 0.76).

##### Differentiation from Other Pulmonary Diseases

**Figure 3:**
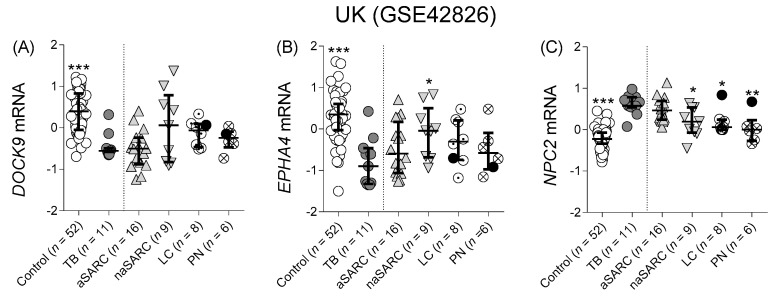
Individual normalized mRNA expression values of (**A**)*DOCK9, (**B**) EPHA4,* and (**C**) *NPC2* mRNA in blood. Diagnostic groups comprise: controls; TB = active tuberculosis; aSARC = active sarcoidosis; naSARC = non-active sarcoidosis; LC = lung cancer; PN = pneumonia. The Kruskal–Wallis test was used to assess significance of differences across more than 2 groups, followed by Dunn’s multiple comparison tests correction. * *p*-value < 0.05; ** *p*-value < 0.01; *** *p*-value < 0.005 with respect to TB. ● single LC and PN patients presenting an *NPC2^high^* profile. UK = United Kingdom.

Using the available datasets from the United Kingdom (GSE42826), we evaluated the potential of the three mRNAs to discriminate between TB and other lung diseases that are likely to constitute clinically important confounders. This study generated whole blood microarray transcriptional data from patients with TB or OD and from controls (Figure 3).

Similarly to the observation in Section *Studies Comparing with Control and LTBI*, in the British cohort, *DOCK9* and *EPHA4* expression was also significantly lower (*p* ≤ 0.0001) in TB patients compared to control (Figure 3A,B). However, only *NPC2* expression levels were significantly higher in TB than in the majority of the other lung diseases, such as non-active sarcoidosis (*p* = 0.021), lung cancer (*p* = 0.018), and pneumonia (*p* = 0.006) (Figure 3C).

Similarly to TB, but on a smaller magnitude, patients with active sarcoidosis had significantly higher blood levels of *NPC2* in comparison with control (*p* < 0.0001), which was not observed for any of the other disease groups (Figure 3C, control vs. non-active sarcoidosis: 0.07, vs. lung cancer: 0.052, vs. pneumonia > 0.99). In fact, TB induced the highest median *NPC2* blood levels (0.58, 95% CI 0.37–0.80), which was followed by active sarcoidosis (0.46, 95% CI 0.23–0.71). Lower median values were observed in non-active sarcoidosis (0.19, 95% CI 0.091–0.54), lung cancer (0.063, 95% CI 0.0018–0.84), and pneumonia (0.00109, 95% CI 0.34–0.675) and the lowest in the control group (−0.22, 95% CI 0.296–0.15). Thus, higher *NPC2* levels in peripheral blood might underlie immunopathological processes that are similar in active sarcoidosis and in TB, but are less common during the cessation of sarcoidosis symptoms, and even lower in lung cancer and pneumonia.

In general, *DOCK9* and *EPHA4* (Figure 3; Table 2, mean AUROC ≤ 0.65) showed lower potential to differentiate between TB and these clinical confounders when compared to *NPC2* (Table 2, mean AUROC = 0.73). All mRNAs had low AUROC values for the distinction TB vs. active sarcoidosis (Table 2, AUROC between 0.51 and 0.69). In contrast, a moderate to high potential to discriminate TB from non-active sarcoidosis (AUROC = 0.87), lung cancer (0.86), and pneumonia (0.88) was observed. In fact, only one patient diagnosed with lung cancer and one with pneumonia showed an *NPC2^high^* profile (black dots, Figure 3C).

### 3.2. Prospective Studies: Group Comparisons and ROC Analysis

#### 3.2.1. Disease Progression

Our previous observation of a subgroup of LTBI subjects with *NPC2* TB-like expression profile [10] raised the question whether this would indicate progression of sub-clinical cases.

**Figure 4:**
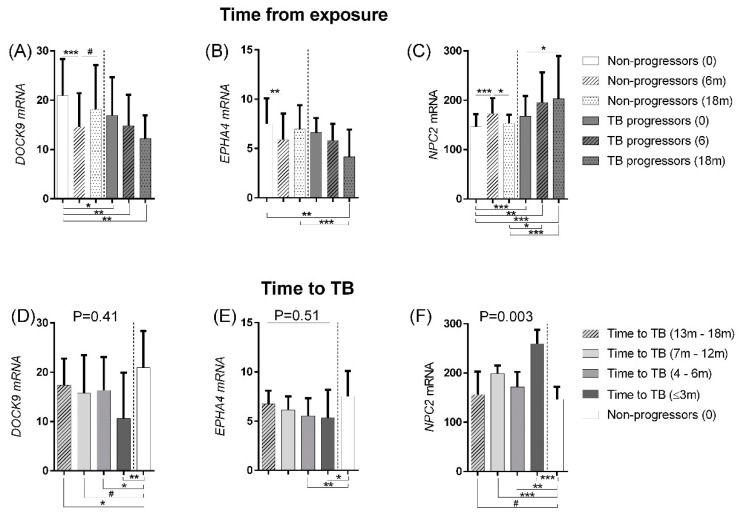
Changes in *DOCK9, EPHA4,* and *NPC2* expression during progression to active TB. Median values (interquartile ranges). The same samples were arranged according to the time from exposure to index case (**A**–**C**) or time to development of active TB (**D**–**F**). Expression values were obtained from the dataset GSE94438. The Mann–Whitney test was used to assess significance between two groups. The Kruskal–Wallis test was used to assess significance of global differences across more than two groups, followed by Dunn’s multiple comparison tests correction. Small bar: *p*-value comparing groups. # *p*-value < 0.1; * *p*-value < 0.05; ** *p*-value < 0.01; *** *p*-value < 0.005.

The Pan African cohort comprises a series of samples collected from household contacts after a person who was recently diagnosed with TB returned to the household (GSE94438). Based on samples collected three times at intervals of 6 months, subjects who developed TB are here classified as “TB progressors” (*n* = 64), and those who did not develop TB in the 18-month window are classified as “non-progressors” (*n* = 208) [24]. At the time of Mtb exposure (time 0), the TB progressors already show differential expression of *DOCK9* and *NPC2* in comparison to the non-progressors (Figure 4A–C, small clades under the graph; *p* < 0.026). During follow up, non-progressors and TB progressors initially showed similar expression changes until month 6, i.e., down-regulation of *DOCK9* (compare white and grey bars, Figure 4A) and *EPHA4* (Figure 4B) and up-regulation of *NPC2* (Figure 4C). Interestingly, after month 6 of exposure, expression of all three mRNA showed a trend toward normalization in non-progressors, although a significant difference was only observed for *NPC2* (as indicated by the significant down-regulation between the dashed and dotted white bars in Figure 4C). In contrast, *NPC2* mRNA levels in TB progressors continued to increase throughout the follow-up period (Figure 4C, compare white and grey bars).

In order to understand the dynamics of transcriptional changes from the time of infection to disease onset, we performed additional analyses on the Pan African GSE94438 dataset. They were grouped into TB progressors, according to the time elapsed between the blood collection and the diagnosis of TB (T<3 m = <3 m, T4–6m = 4–6 m, T7–12m = 7–12 m, or T13–18 m = 13–18 m) and non-progressors at enrollment (time 0) for comparison. This analysis showed that TB progressors already had a significantly lower expression of *DOCK9* at T13–18m before disease development (Figure 4D). Significant up-regulation of *NPC2* among TB progressors was observed from T7 to 12m (Figure 4F), while *EPHA4* showed later expression changes at T4–6m (Figure 4E).

We expected that these expression changes would be more pronounced with decreasing time to disease onset. Indeed, a median decrease in *DOCK9* (Figure 4D) and *EPHA4* (Figure 4E) was observed in individuals with time-to-disease of 3 and 6 months, but only *NPC2* up-regulation was significantly increased up to one year of time-to-disease, with the highest expression observed in individuals with the shortest time-to-disease (3 months) (*p* = 0.003; Figure 4F, dark grey bars). Altogether, these findings suggest that monitoring *NPC2* expression in blood might serve as biomarker for progression to TB among individuals recently exposed to Mtb or among household contacts of recently diagnosed TB cases.

#### 3.2.2. Correlation with Completion of Anti-TB Treatment

Besides their potential to detect disease cases, expression of optimal biomarkers for TB should reflect successful completion of anti-TB treatment. To evaluate this aspect, we used two datasets, China GSE54992 [25] and South Africa GSE89403 [7], which featured prospective sampling during anti-TB treatment. The dataset GSE54992 comprises microarray expression data from PBMC, and GSE89403 is an RNAseq-based study of whole blood.

**Figure 5:**
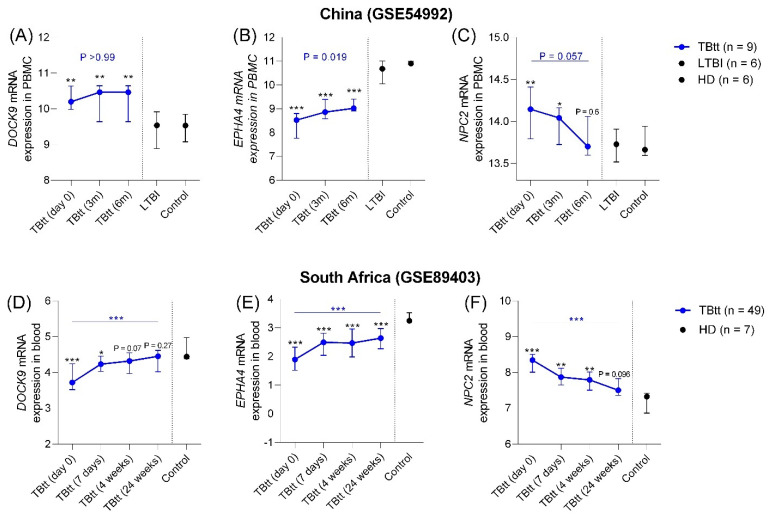
Effects of anti-tuberculosis chemotherapy on the expression of *DOCK9*, *EPHA4,* and *NPC2* mRNAs in blood samples from TB patients. Transcriptomic data were obtained from two publicly available datasets, China (GSE54992, **A**–**C**) and South Africa (GSE89403, **D**–**F**). Follow-up samples from TB-treated (TBtt) patients were collected before treatment initiation (day 0), at different time intervals during therapy (GSE54992: 3 months [m]; GSE89403: 7 days, and 1 month), and at the end of therapy (GSE54992: and GSE89403: 6 months). The Friedman test was used to assess significance of the longitudinal analysis among TB cases during treatment. The Mann–Whitney test was used to assess significance between two groups vs. LTBI or vs. control. The Kruskal–Wallis test was used to assess significance of global differences across more than two groups, followed by Dunn’s multiple comparison tests correction. *p*-values obtained comparing TBtt time intervals against non-TB cases (LTBI and control) are shown in black (●). *p*-values obtained comparing expression changes in TBtt patients during treatment are shown in blue (●). * *p*-value < 0.05; ** *p*-value < 0.01; *** *p*-value < 0.005.

In accordance with the cross-sectional evaluation shown in Figure 1 and Figure 2, in this section, significant differences in *DOCK9, EPHA4*, and *NPC2* expression were also evident in all pairwise comparisons between untreated active TB patients (TB) and LTBI and controls (see Figure 5, *p*-values in black font). However, although *DOCK9* was down-regulated in the previous analyses, it was up-regulated in PBMC from TB cases in the Chinese dataset (Figure 5A). Apart from this discrepancy, significant *DOCK9^low^* (GSE89403 only; *p* < 0.0001), *EPHA4^low^* (GSE54992 and GSE89403; *p* < 0.001) and *NPC2^high^* (GSE54992 and GSE89403; *p* ≤ 0.03) expression differences in the comparison TB vs. control/LTBI were also observed among these datasets from China and South Africa.

During anti-TB chemotherapy, expression of these three genes changed significantly over time. Even though expression of all three tended to normalize, it is notable that only *NPC2* expression levels did not differ between control/LTBI groups and TBtt by the end of therapy in both cohorts (Figure 5C [control/LTBI vs. TBtt (6 m), *p* = 0.6] and 5F [control vs. TBtt (24 weeks), *p* = 0.096]). In the South African cohort, *DOCK9* also showed a gradual normalization and no statistically different expression levels between control and TBtt at the end of therapy.

Moreover, expression data available from the Haitian cohort after two weeks of anti-TB treatment (Figure S1) corroborated the significant reduction in *NPC2* mRNA blood levels even in this early stage of anti-TB treatment.

### 3.3. NPC2 Accuracy: Sensitivity and Specificity Analysis

Overall, in the previous sections, *NPC2* showed better discriminatory potential for TB across the different group comparison analyses. Therefore, we proceeded with a more detailed ROC curve analysis exclusively for *NPC2*.

As the selection of cut-off values can be adjusted in order to improve either the sensitivity or the specificity of a given test, we decided to assess *NPC2* accuracy in various possible diagnostic scenarios. For this purpose, we calculated its sensitivity and specificity for the detection of TB according to: (i) maximum Youden index; (ii) the TPP for a community-based triage or referral test to identify people suspected of having TB (9); (iii) the TPP for a test for predicting progression from TB infection to active disease (12).

As shown in Table 3, analyses performed at the maximum Youden index (sensitivity + specificity/2) showed high mean sensitivities for TB detection varying between 87.5% and 100% vs. controls and moderate values when compared to LTBI (72.7–75%), while exhibiting high mean specificity (90.2–100%). The analysis comparing TB against OD showed the lowest mean specificity in the case of TB vs. active sarcoidosis (56.3%), but for the other TB clinical confounders, these values were ≥79%, maintaining a sensitivity of ≥72.7%. However, discriminatory power decreased when comparing TB vs. S-NTB, as sensitivity and specificity were ≤67.8%.

By adjusting the accuracy analysis to meet the TPP for a community-based triage or referral test to identify people suspected of having TB, we observed that most of the analyses meet the minimum sensitivity (>90%) and specificity (>70%) requirements (Table 3, bold font). Only the cohorts from Brazil and South Africa (in which the control and S-NTB groups were composed of recent close contacts or symptomatic respiratory patients, respectively) and comparisons between TB and naSARC and aSARC did not meet this TPP minimum criteria.

In contrast, it is noteworthy that in the longitudinal analysis, *NPC2^high^* fulfilled the minimum TPP sensitivity and specificity (>75%) criteria for a monitoring test for prediction of progression from latent to active TB [19]. Here, *NPC2^high^* could detect subjects that will progress to active TB in a time interval <3 m before disease onset (Pan African cohort GSE94438, see bold numbers in Table 3), but not later than that. If we aim for a maximum detection of TB cases, i.e., higher sensitivity, *NPC2^high^* demonstrated 92.3% mean sensitivity and 75% mean specificity to detect TB < 3 m before disease onset.

## 4. Discussion

We have performed an external validation/re-evaluation of the mRNA triplet *DOCK9*, *EPHA4*, and *NPC2*, which we had previously identified as potential biomarkers in whole blood of Brazilian TB patients [10]. We observed similar changes in expression among subjects from different countries, regardless of differences in genetic background and local TB incidence ( Figure 1 and Figure 2, Table 2). Our results suggest that the gene set was differentially expressed among patients with active TB and that *NPC2^high^* should perform better even in high burden areas such as South Africa (520/100.000), India (199/100.000), and Haiti (176/100.000). Notably, the single microarray dataset included in this study (GSE42826) confirmed the differential expression of the three genes originally uncovered by RNAseq, corroborating a previous study showing the equivalence of microarray and RNAseq to assess differential gene expression [29].

Our study, which was done in different cohorts, shows mean AUROC values > 0.90 of *NPC2* for the detection of active TB, which was consistent in all cohorts for the discrimination between TB cases and control, with the highest mean AUROC values compared to *EPHA4* and *DOCK9*. In addition, *NPC2* expression was significantly lower in other lung diseases, except for active sarcoidosis (Figure 3). Even though mycobacteria and propionibacteria are the most commonly implicated etiologic agents of sarcoidosis, based on studies using PCR amplification of microbial DNA, so far, *Propionibacterium acnes* is the only microorganism successfully isolated from sarcoid lesions by bacterial culture, which may help in the diagnostic differentiation from TB [30,31,32,33].

The biomarker reproducibility of a signature with a small number of genes may be a concern, as some studies have reported that increasing the number of genes may improve sensitivity and specificity [26]. Yet, other studies have reported that a signature comprising 16 genes, which initially predicted progression from LTBI to TB, decreased in accuracy during external validation, failing in the validation in cohorts from other countries [24]. More recently, a systematic review evaluated the accuracy of several proposed transcriptional signatures in a setting with a high burden of TB and HIV in South Africa [6]. Note that none of 27 selected signatures met the WHO optimum or minimum criteria for triage (95% sensitivity and 80% specificity) or confirmatory test (65% sensitivity and 98% specificity), including *NPC2* [10]. In contrast, in the present study, especially *NPC2* demonstrated high mean sensitivity (>87.5%) to distinguish between TB and LTBI, even though a “TB-like” expression profile was also observed among some subjects classified as LTBI and, more frequently, among S-NTB. It is also important to mention that the study by Turner et al. [6] did not feature a confirmatory analysis of these genes, whereas differential expression of *NPC2* in TB was already confirmed by (RT) qPCR in a different Brazilian cohort not included in the present study [10]. Additionally, a diagnostic algorithm tree combining *NPC2* expression cut-off values combined with the results provided by the low-cost chest X-ray examination enabled accurate discrimination between TB and LTBI individuals [10]. When applied in a population to be prospectively evaluated for TB, this type of holistic approach combining imaging findings with transcriptional signatures should be considered in future studies.

Furthermore, we also found that *NPC2^high^* met the minimum sensitivity and specificity TPP criteria for predicting progression from latent tuberculosis to active TB in most of the cohorts. The lower sensitivity/specificity observed for the comparisons with the South African respiratory symptomatic cohort (E-MTAB-8290, Table 3) might seem to be a drawback at first. However, we have to consider the limitations of the current diagnostic tests for TB detection [2,3,4,5] and that an *NPC2*^high^ pattern may already be observed at earlier stages of the TB progression spectrum (as seem on Figure 4F). Thus, S-NTB individuals showing an *NPC2*^high^ profile in blood could be harboring a sub-clinical/paucibacillary TB infection.

We identified *NPC2* as an accurate marker for identifying individuals at high risk of progressing from LTBI to TB. If the sensitivity of 92.3% and specificity of 75% (Table 3) for predicting progression from latent to active TB is corroborated in additional studies, monitoring *NPC2* expression in blood can contribute to the detection and early treatment of those LTBI cases at risk of progression to active TB by using a simpler method, (RT) qPCR, which was already validated for this marker [10]. In addition, chest X-ray would be an easy tool to perform in order to screen high-risk LTBI vs. active TB cases among subjects with an *NPC2^high^* profile. On the other hand, considering that all-trans retinoic acid (ATRA) triggers an *NPC2*-dependent antimicrobial response against Mtb [18], it is important to investigate whether vitamin A deficiency could contribute to false-negative results in individuals otherwise expected to have an *NPC2^high^* pattern. Clearly, there is a need for additional prospective studies to validate our current findings.

Our data of non-progressor household TB contacts showed significant changes in *NPC2* gene expression only 6 months after exposure to the initial index case, with a return of expression to the initial level after 18 months (Figure 4). However, a different dynamic was observed for the TB progressors, which showed a continual up-regulation of this gene expression toward the TB profile with increasing proximity to disease onset (Figure 4F). Nowadays, TST and IGRA are the eligible tests to identify *M. tuberculosis* exposure, although they cannot distinguish TB vs. LTBI or identify individuals who will progress from latent to active TB within the next two years [5]. These immune response-based tests do not reflect the presence of live bacilli in the host and are still positive after completion of treatment of the infection. As exemplified by *NPC2* mRNA, this drawback can be overcome by measuring the expression of genes that play a functional role in host defenses against the pathogen. Measuring its activity at the mRNA level may be a particularly attractive option due to the highly dynamic nature of transcriptional responses in the host’s biological processes [34]. The sensitivity and high dynamic range of many transcriptomic responses likely also explains the ability of *NPC2* to predict progression to TB and its correlation with treatment completion.

TB control critically relies on the identification of individuals with active disease and the administration of complete drug treatment. However, to follow the response to treatment, the tools available, such as in vitro culture of clinical specimens, are slow and laborious. Rapid molecular tests, such as GeneXpert^®^, as well as the less expensive sputum stain for acid-fast bacilli, may produce false positive results due to the detection of residual nucleic acids and structure of dead bacilli, respectively [35]. For the South African cohort, we observed a significant decrease (*p* = 0.0037, Figure 5F) in *NPC2* levels during treatment, with a borderline significance (*p* = 0.057, Figure 5C) for the Chinese cohort. The presence of drug-resistant strains can affect the outcome of anti-TB treatments and could explain the borderline significance observed on Figure 5C (blue line). Unfortunately, we cannot do any further analysis in this regard, since information on the presence of drug resistance is not available for these cohorts.

## 5. Conclusions

In summary, this analysis of publicly available datasets from different geographic areas validates our previous findings that *NPC2* is a promising host biomarker for diagnosing TB. Potential use as a differential diagnostic between TB and other lung diseases was also observed, although the diagnostic performance was slightly lower among subjects from South Africa with respiratory symptoms. Notably, we obtained additional evidence indicating that up-regulation of this gene in blood might also be used for predicting progression from latent to active infection (also fitting to the minimum TPP criteria from WHO) and for monitoring response to anti-TB treatment. The relatively low number of subjects in the independent validation cohorts is an important limitation of this study. Further studies are required to corroborate our findings, including heterogeneous cohorts with larger sample sizes, different TB clinical confounders, and doing prospective evaluations during disease progression and anti-TB treatments.

## Figures and Tables

**Table 1:** Overview of the included cohorts.

Study	Publication Year	Study Country	Method	Diagnostic Groups						Public ID	Reference
				Control	S-NTB	LTBI	TB	TBtt	OD		
*Cross-sectional*	2016 2019	Brazil	RNAseq	14 ^d^	-	21 ^e^	8 ^f^	-	-	GSE84076 GSE131174	[10] [11]
	2019	Haiti	RNAseq	14	-	41	22	-	-	Not deposited	[20,21]
	2017	India	RNAseq	-	-	28	16	-	-	GSE101705	[26]
	2012	UK	Microarray	52	-	-	11	-	39 ^c^	GSE42826	[23]
	2020	SA	RNAseq	-	127	-	54	-	-	E-MTAB-8290	[6]
	2017	Pan A	RNAseq	208 ^a^	-	-	64 ^b^	-	-	GSE94438	[24]
*Prospective*	2014	China	Microarray	6	-	-	6	9	-	GSE54992	[25]
	2016	SA	RNAseq	7	-	-	-	49	-	GSE89403	[7]

All studies used whole blood as clinical specimen except the Chinese cohort, which worked with PBMC. ^a^ Non-progressors to active TB during the period of the respective study. ^b^ Progressors to active TB during the period of the respective study. ^c^ Other respiratory diseases (OD) comprising active (*n* = 16) or non-active sarcoidosis (*n* = 9), lung cancer (*n* = 8), and pneumonia (*n* = 6). ^d,e,f^ contain 12, 14, and 6 samples previously analyzed in de Araujo et al. 2016, respectively. SA = South Africa, UK = United Kingdom, Pan A = Pan-African. Specimen collection and RNA isolation.

**Table 2:** ROC statistics.

Study	Platform	Age Group	Study Site	TB Incidence ^¥^	Study Period (Month/Year)	Specimen	Reference Negative (n)	Reference Positive (n)	AUROC (95% CI)		
									*DOCK9*	*EPHA4*	*NPC2*
de Araujo et al	RNAseq	Adults	Brazil	45	03/2010–08/2013	Whole blood	Control (14)	TB (6)	0.86	0.89	0.94
									(0.70 to 1.0)	(0.76 to 1.0)	(0.81 to 1.0)
							LTBI (21)		0.86	0.91	0.94
									(0.71 to 1.0)	(0.80 to 1.0)	(0.83 to 1.0)
Haiti	RNAseq	Adults	Haiti	176	02/2016–08/2020	Whole blood	Control (14)	TB (22)	0.96	0.97	0.91
									(0.89 to 1.0)	(0.92 to 1.0)	(0.79 to 1.0)
							IGRApos (41)		0.95	0.96	0.89
									(0.89 to 1.0)	(0.92 to 1.0)	(0.80 to 0.98)
GSE101705	RNAseq	Adults	India	199	NA	Whole blood	LTBI (28)	TB (16)	0.92	0.93	0.98
									(0.84 to 1.0)	(0.85 to 1.0)	(0.93 to 1.0)
GSE42826	Microarray	Adults	United Kingdom	8	09/2009–03/2012	Whole blood	Control (52)	TB (11)	0.93	0.90	0.99
									(0.84 to 1.0)	(0.79 to 1.0)	(0.97 to 1.0)
							aSARC (16)		0.51	0.69	0.66
									(0.29 to 0.74)	(0.47 to 0.90)	(0.45 to 0.87)
							naSARC (9)		0.61	0.80	0.87
									(0.31 to 0.91)	(0.60 to 1.0)	(0.71 to 1.0)
							LC (8)		0.88	0.75	0.86
									(0.71 to 1.0)	(0.53 to 0.97)	(0.65 to 1.0)
							PN (6)		0.71	0.68	0.88
									(0.41 to 1.0)	(0.42 to 0.94)	(0.68 to 1.0)
Mean (all of the above)							Control (80)	TB (55)	0.92	0.92	0.95
									(0.79 to 1.0)	(0.81 to 1.0)	(0.84 to 1.0)
							LTBI (90)		0.91	0.93	0.94
									(0.87 to 0.99)	(0.87 to 1.0)	(0.83 to 1.0)
							OD (37)		0.65	0.63	0.73
									(0.50 to 0.80)	(0.62to 0.79)	(0.56 to 0.90)

^¥^ Cases per 100,000 inhabitants/year, source: https://www.who.int/tb/country/data/profiles/en/; diagnostic groups are composed of controls, latent tuberculosis infection (LTBI), and active tuberculosis (TB). aSARC = active sarcoidosis; naSARC = non-active sarcoidosis; LC = lung cancer; PN = pneumonia; NA = not available.

**Table 3:** Accuracy of NPC2 transcription s following WHO´s target product profile (TPP) for blood biomarkers for TB.

Study or GEO Accession Number	Country	Comparison		Sensitivity and Specificity Analysis Adjusted to:									
				Maximum Youden Index		TPP for a Community-Based Triage or Referral Test to Identify People Suspected of Having TB ^a^				TPP for a Test for Predicting Progression from TB Infection to Active Disease ^a^			
						Minimum SENSITIVITY: ≥90%		Minimum SPECIFICITY: ≥70%		Minimum SENSITIVITY: ≥75		Minimum SPECIFICITY: ≥75	
				Sensitivity (95% CI)	Specificity (95% CI)	Sensitivity (95% CI)	Specificity (95% CI)	Sensitivity (95% CI)	Specificity (95% CI)	Sensitivity (95% CI)	Specificity (95% CI)	Sensitivity (95% CI)	Specificity (95% CI)
***Cross-sectional analyses***													
***Control or LTBI vs TB***													
de Araujo et al 2016	BR	Control (*n* = 14) ^b^	vs TB (*n* = 6)	87.5 (47.4–99.7)	100 (76.8–100)	100 (63.1–100)	50 (23–77)	87.5 (47.4–99.7)	100 (76.8–100)	**87.5** ** (47.4–99.7)**	**100** **(76.8–100)**	**87.5** ** (47.4–99.7)**	**100** ** (76.8–100)**
		LTBI (*n* = 21)		75 (34.9–96.8)	100 (83.9–100)	100 (63.1–100)	57.1 (34–78.2)	87.5 (47.4–99.7)	90.5 (69.6–98.9)	**75** **(34.9–96.8)**	**100** **(83.9–100)**	**87.5** **(47.4–99.7)**	**90.5** **(69.6–98.8)**
Haiti	H	Control (*n* = 14) ^c^	vs TB (*n* = 22)	90.9 (70.8–98.9)	100 (75.3–100)	**90.9** **(70.8–98.9)**	**100** **(75.3–100)**	**95.5** **(77.2–99.9)**	**76.9** **(46.2–95)**	**90.9** **(70.8–98.9)**	**100** **(75.3–100)**	**95.5** **(77.2–99.9)**	**76.9** **(46.2–95)**
		LTBI (*n* = 41)		72.7 (49.8–89.3)	90.2 (76.8–97.3)	**90.9** **(70.8–98.9)**	**78.1** **(62.4–89.4)**	**90.9** **(70.8–98.9)**	**70.7** **(54.5–83.9)**	**77.3** **(54.6–92.2)**	**87.8** ** (73.8–95.9)**	**90.9** **(70.8–98.9)**	**78.1** **(62.4–89.4)**
GSE101705	I	LTBI (*n* = 28)	vs TB (*n* = 16)	96.4 (81.7–99.9)	100 (79.4–100)	**96.4** ** (81.7–99.9)**	**100** **(79.4–100)**	**96.4** **(81.7–99.9)**	**100** **(79.4–100)**	**96.4** **(81.7–99.9)**	**100** ** (79.4–100)**	**96.4** **(81.7–99.9)**	**75** **(47.6–92.7)**
***OD vs TB***													
GSE42826	UK	Control (*n* = 52) ^c^	vs TB (*n* = 11)	100 (71.5–100)	90.4 (79–96.8)	**90.9** ** (58.7–99.8)**	**96.2** **(86.8–99.5)**	**100** **(71.5–100)**	**71.2** **(56.9–82.9)**	**81.8** **(48.2–97.7)**	**100** **(93.2–100)**	**100** **(71.5–100)**	**75** **(61.1–86)**
		aSARC (*n* = 16)		81.8 (48.2–97.8)	56.3 (29.9–80.3)	90.9 (58.7–99.8)	43.8 (19.8–70.1)	45.5 (16.8–76.6)	75 (47.6–92.8)	81.8 (48.2–97.7)	56.3 (29.9–80.3)	45.5 (16.8–76.6)	75 (47.6–92.7)
		naSARC (*n* = 9)		72.7 (39–94)	88.9 (51.8–99.7)	90.9 (58.7–99.8)	66.7 (29.9–92.5)	81.8 (48.2–97.7)	77.8 (40–97.2)	**81.8** ** (48.2–97.7)**	**77.8** **(40–97.2)**	**81.8** ** (48.2–97.7)**	**77.8** **(40–97)**
		LC (*n* = 8)		81.8 (48.2–97.7)	87.5 (47.4–99.7)	**90.9** ** (58.7–99.8)**	**87.5** ** (47.4–99.7)**	**90.9** ** (58.7–99.8)**	**75** ** (34.9–96.8)**	**81.8** **(48.2–97.7)**	**87.5** ** (47.4–99.7)**	**90.9** ** (58.7–99.8)**	**87.5** **(47.4–99.7)**
		PN (*n* = 6)		90.9 (58.7–99.8)	83.3 (35.9–99.6)	**90.9** **(58.7–99.8)**	**83.3** ** (35.9–99.6)**	**90.9** ** (58.7–99.8)**	**83.3** ** (35.9–99.6)**	**81.8** **(48.2–97.7)**	**87.5** **(47.4–99.7)**	**90.9** **(58.7–99.8)**	**83.3** **(35.9–99.6)**
***Mean***													
All of the above		Control (*n* = 80) ^c^	TB (*n* = 55)	92.8 (76.8–100)	96.8 (83–100)	**93.9** **(80.9–100)**	**82.1** **(12.9–100)**	**94.3** ** (78.6–100)**	**82.7** **(44.8–100)**	**86.7** **(75.3–98.2)**	**100** **(100–100)**	**94.3** ** (78.6–100)**	**84** **(49.4–100)**
		LTBI (*n* = 90)		81.4 (48.9–100)	96.7 (82.7–100)	**95.8** **(84.4–100)**	**78.4** ** (25.1–100)**	**91.6** **(80.4–100)**	**87.1** ** (49.9–100)**	**82.9** ** (53.7–100)**	**95.9** ** (78.4–100)**	**91.6** ** (80.4–100)**	**81.2** **(60.8–100)**
		OD (*n* = 39)		81.8 (70–93.6)	79 (54.6–100)	90.9 (90.9–90.9)	70.3 (38.8–100)	77.3 (42.9–100)	77.8 (71.6–84)	NA	NA	NA	NA
***Symptomatic respiratory***													
E-MTAB-8290	SA	S-NTB (*n* = 127)	TB (*n* = 54)	61.1 (46.9–74.1)	67.8 (58.9–75.7)	90.7 (79.7–96.9)	26 (18.6–34.5)	57.4 (43.2–70.8)	70 (61.3–77.9)	75.9 (62.4–86.5)	49.6 (40.6–58.6)	64.81 (50.6–77.3)	91.3 (85.0–95.6)
***Prospective analyses***													
GSE94438	PA	Control (*n* = 208)	vs TB_(≤3 m)_ (*n* = 13)	92.3 (64–99.8)	83.2 (77.4–88)	**92.3** ** (64–99.8)**	**83.2** **(77.4–88)**	**100** **(75.3–100)**	**74.5** **(68–80.39)**	**76.9** ** (46.2–95)**	**87.5** ** (82.2–91.7)**	**92.3** ** (64–99.8)**	**75** ** (68.5–80.7)**
		Control (*n* = 208)	vs TB_(4–6m)_ (*n* = 34)	76.5 (58.8–89.3)	53.9 (46.8–60.8)	91.2 (76.3–98.1)	23.1 (17.5–29.4)	52.9 (35.1–70.2)	70.2 (63.5–76.3)	76.5 (58.8–89.3)	53.9 (46.8–60.8)	50 (32.4–67.6)	75 (68.5–80.7)
		Control (*n* = 208)	vs TB_(7–12m)_ (*n* = 19)	57.9 (33.5–79.8)	90.4 (85.5–94)	94.7 (74–99.9)	31.3 (25–38)	63.2 (38.4–83.7)	76 (69.6–81.6)	79 (54.4–94)	61.1 (54.7–67.7)	63.2 (38.4–83.7)	75 (68.5–80.7)
		Control (*n* = 208)	vs TB_(13–18m)_ (*n* = 32)	84.4 (67.2–94.7)	44.7 (37.8–51.7)	90.6 (75–98)	30.3 (24.1–379)	40.6 (23.7–59.4)	70.2 (63.5–76.3)	75 (56.6–88.5)	50 (43–57)	34.4 (18.6–53.2)	75 (68.5–80.7)

Diagnostic groups are composed of exposed controls, latent tuberculosis infection (LTBI) and active tuberculosis (TB). S-NTB = symptomatic non-TB; aSARC = active sarcoidosis; naSARC = non-active sarcoidosis; LC = lung cancer; PN = Pneumonia. BR = Brazil, H = Haiti; I = India; UK = United Kingdom; PA = Pan African (SA, the Gambia, Ethiopia, and Uganda). ^a^ Results that fulfilled the TPP minimum sensitivity and specificity recommendations are printed in bold. ^b^ Healthy individuals recently exposed to a TB index case. ^c^ Healthy individuals with no known recent contact with a TB index case. NA = non-applicable.

## Data Availability

The data supporting the reported results can be found as follows: GSE84076: https://www.ncbi.nlm.nih.gov/geo/query/acc.cgi?acc=GSE84076. GSE131174: https://www.ncbi.nlm.nih.gov/geo/query/acc.cgi?acc=GSE131174. Haiti: https://www.ncbi.nlm.nih.gov/bioproject/PRJNA445968. GSE101705: https://www.ncbi.nlm.nih.gov/geo/query/acc.cgi?acc=GSE101705. GSE42826: https://www.ncbi.nlm.nih.gov/geo/query/acc.cgi?acc=GSE42826. E-MTAB-8290: https://www.ebi.ac.uk/arrayexpress/experiments/E-MTAB-8290/?page=1&pagesize=250. GSE94438: https://www.ncbi.nlm.nih.gov/geo/query/acc.cgi?acc=GSE94438. GSE54992: https://www.ncbi.nlm.nih.gov/geo/query/acc.cgi?acc=GSE54992. GSE89403: https://www.ncbi.nlm.nih.gov/geo/query/acc.cgi?acc=GSE89403.

## Supplementary Materials

The following are available online at https://www.mdpi.com/article/10.3390/cells10102704/s1, Figure S1: Effects of anti-tuberculosis chemotherapy on expression of *DOCK9, EPHA4* and *NPC2* mRNAs in blood specimens from tuberculosis (TB) patients.
