# Supplementary material for: Transcriptomic Biomarkers for Tuberculosis: Validation of NPC2 as a Single mRNA Biomarker to Diagnose TB, Predict Disease Progression, and Monitor Treatment Response
Source: Cells. 2021 Oct 9;10(10):2704. doi: 10.3390/cells10102704 (PMC8534371; doi:10.3390/cells10102704)
Supplement: Supplementary file 1 [file cells-10-02704-s001.zip › figure S1.pdf]

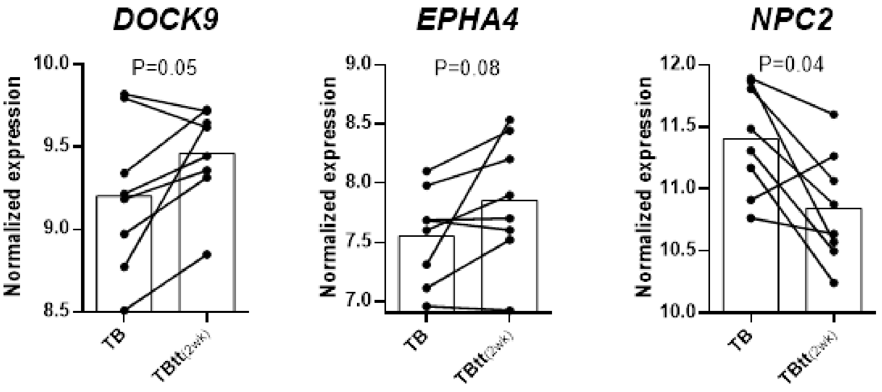

Figure S1 – Effects of anti-tuberculosis chemotherapy on expression of *DOCK9, EPHA4* and *NPC2* mRNAs in blood specimens from tuberculosis (TB) patients. Transcriptomic data from a cohort of Haitian (n = 8) collected at TB diagnosis (time 0) and 2 weeks after the onset of anti-TB chemotherapy ($TBtt_{(2wk)}$). Further follow-up data until the end of anti-TB therapy are not available. P-values by Wilcoxon test.
